# Informing Intervention: An Exploration of Behavioral and Social–Emotional IEP Goals for Students with ASD

**DOI:** 10.3390/bs16030417

**Published:** 2026-03-12

**Authors:** Sarah K. Cox, Courtenay A. Barrett, Goretty Chavez, Rebecca Saur, Megan Feury, Grace Huber, Brianna Booms, Gabrielle Snyder

**Affiliations:** Department of Counseling, Educational Psychology and Special Education, College of Education, Michigan State University, East Lansing, MI 48824, USA; morsicou@msu.edu (C.A.B.);

**Keywords:** autism spectrum disorder, social–emotional learning, behavior, Individualized Education Programs

## Abstract

Students with autism spectrum disorder (ASD) experience impairments in reciprocal social interactions, communication, and a restricted range of interests or repetitive behaviors that impact the development of their behavioral and/or social–emotional skills. In schools, students with ASD receive Individualized Education Programs (IEPs), which include goals to understand the types of behavioral and/or social–emotional skills students are working to develop. However, there is scant empirical research examining the nature of IEP goals that target behavioral and/or social–emotional skills among students with ASD. The current study explores the content, scope, and location of behavioral and social–emotional IEP goals for 153 students with ASD in Grades K-12 in one state in the Upper Midwest. Understanding the nature of IEP goals is a critical first step to increase access to evidence-based behavioral and social–emotional interventions for students with ASD. Implications for school-based behavioral and psychosocial interventions for students with ASD are discussed.

## 1. Introduction

Behavioral and social–emotional skills serve as the foundation for successful participation in academic and social environments (Ruble et al., 2010a; Pennington, 2017). Developing behavioral and social–emotional skills are particularly important for students with ASD, as they address core features of ASD, such as challenges with effective communication, executive functioning, and restrictive behaviors. When IEPs include specific, measurable goals for behavioral and social–emotional growth, students are more likely to experience growth in self-advocacy, resilience, friendship skills, and adaptability—key predictors of success both in and beyond school. Furthermore, Chazin et al. (2025) identified priorities of individuals with ASD and their families, which included (a) decreasing self-injurious behaviors, (b) enhancing self-help skills, (c) effectively using various communication modalities, and (d) identifying emotions in themselves and others. Despite their importance, behavioral and social–emotional goals are often missing or poorly articulated in IEPs for students with ASD, resulting in unmet needs that can affect engagement, learning, and well-being (e.g., Chazin et al., 2025; Humbert et al., 2025; Pennington, 2017; Ruble et al., 2010b). Addressing these areas in educational planning ensures students with ASD receive targeted support and interventions to develop essential life skills, foster healthy relationships, and thrive in inclusive communities.

### 1.1. Role of Individualized Education Programs

Individualized Education Programs (IEPs) serve as the roadmap for every K-12 public school child who receives special education services (Individuals with Disabilities Education Act, 2004). A critical component of the IEP is a list of measurable annual goals to address the individualized needs of each student, along with a plan to monitor the student’s progress toward meeting those goals (Individuals with Disabilities Education Act, 2004). As described in the field of implementation science, measurable annual goals are necessary for IEP teams to select and monitor the effectiveness of educational programming (Odom et al., 2010). High-quality annual goals include (a) a specific description of an observable target behavior, (b) specific conditions under which the student is expected to perform the behavior (e.g., when, where, and with whom), (c) the criterion level and timeline for goal achievement, and (d) a process for measuring student progress toward the goal (Ruble et al., 2010b; Müller et al., 2023; Notari, 1988). These constructs align with federal and state policies (e.g., Individuals with Disabilities Education Act, 2004; Michigan Administrative Rules for Special Education, 2019) indicating all IEP goals include (a) a student’s current level of performance, (b) the specific skill to be measured, (c) the target or outcome, and (d) the method of measurement. Poorly defined goals contribute to challenges in assessing student progress, leading to missed opportunities for targeted intervention and adjustment (Odom et al., 2010). Focusing on IEP goals as a key component of the full IEP document increases the feasibility of conducting high-quality research and provides adequate information to inform practice, while minimizing risks to schools, students, and families, which may be introduced related to privacy or confidentiality by analyzing other components (e.g., services provided).

### 1.2. Literature Review

#### 1.2.1. Measuring the Quality of IEP Goals

Given the importance of high-quality IEP goals for students with ASD, several researchers have investigated methods for determining if goals are specific, measurable, and actionable. Ruble et al. (2010b) proposed rigorous criteria for high-quality IEP goals, emphasizing the necessity for goals to clearly specify the learner, the context or condition under which a behavior is expected, the observable behavior itself, and measurable criteria to evaluate attainment. The IEP evaluation tool (IEP-Q), validated by Ruble et al. (2010b), provides researchers and educators with a rubric to evaluate the *full* IEP document for young children with ASD, but may be less useful when the *full* IEP is not necessary or not available. The IEP-Q includes eight indicators of goal quality, providing the opportunity to employ more nuanced tools that capture a greater number of quality indicators or characteristics, by disaggregating where, when, and by whom the goal will be assessed. Further, examining IEP goals separately from the full IEP may enable researchers to gain access to otherwise unavailable data to answer specific research questions (e.g., schools may be unwilling or unable to share the full document). The Revised IEP/Individualized Family Service Plan (IFSP) Goals and Objectives Rating Instrument (R-GORI; Notari, 1988) has been utilized by others to assess the quality of IEP goals for students with speech and sound disorders (Farquharson et al., 2014), traumatic brain injury (Goodwin et al., 2022), and for preschoolers with disabilities (Rakap, 2015). However, given the broad scope of the R-GORI for a variety of populations (Notari, 1988), most researchers chose to modify the instrument by not including components that were irrelevant for their context, and/or supplementing the tool with contextualized information to ensure the coding system was sensitive enough to capture outcomes of interest (e.g., Humbert et al., 2025). Recommendations for assessing goal quality consistently emphasize the importance of (a) specific, measurable, and observable behaviors, (b) conditions or context under which the behavior is expected, and (c) a method for capturing qualitative and/or quantitative data to measure student progress (Hott et al., 2021; Humbert et al., 2025, Ruble et al., 2010b).

#### 1.2.2. Quality of IEP Goals

Few studies have examined the quality of IEP goals for students with ASD, although this body of research consistently indicates the quality of IEP goals for students with ASD or other disabilities in authentic school contexts often falls short of best practice standards (Müller et al., 2023). For example, Ruble et al. (2010b) found fewer than 10% of goals included quantitative criteria to monitor students’ progress, and around 40% of the goals included a specific, measurable behavior. However, this study included a small sample size of 35 teachers of 39 students with ASD, ages 3 to 9, in two states (one in the Midwest, one in the South). More than ten years later, Humbert et al. (2025) found goal measurability (especially describing conditions/context) had improved in their sample (*N* = 73 students with ASD), compared to the Ruble et al. (2010b) sample, although they noted that the overall IEP quality ratings remained poor for students with ASD. Humbert et al. (2025) hypothesized the R-GORI may not have been sensitive enough to capture quality differences and recommended additional research on IEP quality for students with ASD. Finally, Hott et al. (2021) further documented that many IEP goals for students with social, emotional, or behavioral needs due to a specific learning disability in mathematics lacked completeness, with a significant proportion missing critical components such as clear conditions or measurable progress indicators—reducing their utility in guiding instruction, intervention, and monitoring outcomes. Without a measurable and measured IEP goal, Christle and Yell (2010, p. 112) suggest the IEP is “inadequate.” Therefore, this study contributes to the literature by examining the IEP goals of 153 students with ASD, over twice the sample size included in prior research.

#### 1.2.3. Content of IEP Goals

Very few studies have examined the content of IEP goals for students with ASD. The domains targeted within IEP goals reveal both strengths and gaps in addressing the comprehensive needs of students with ASD. Research by Humbert et al. (2025), Findley et al. (2022), and McIntyre and Zajic (2025) found that while social skills are the most frequent goal type, critical areas such as behavioral regulation and communication skill development are not uniformly included or prioritized. For example, in a survey of 551 primary caregivers, McIntyre and Zajic (2025) found the most common goal area reported to be included on IEPs for students with ASD was social skills (81%), and the least common goal area was behavioral (57%). Furthermore, Humbert et al. (2025) reported that while IEPs for young children with ASD mostly contained goals related to core areas of need (e.g., communication skills, social skills, learning/work skills), social skill goals were absent for 40% of the students, and goals related to alternative/augmented communication were absent for 30% of students who were described as not using verbal communication. This study builds on prior research by examining IEP goals for a larger sample of students, using a Likert-type scale to capture nuanced differences.

By systematically examining the domains represented in active IEPs for students with ASD (e.g., emotional regulation, self-injurious behaviors, executive functioning, alternative/augmentative communication, pragmatic language) educators and researchers can identify gaps in current practice, assess alignment with stakeholder priorities, and better understand the needs of diverse learners. This information can guide the development of targeted interventions, resource allocation, and professional development priorities to increase the effectiveness and relevance of school-based supports. Additionally, investigating the content of IEP goals provides a foundation for future studies on the effectiveness and social validity of specific interventions, helping to ensure that educational programming remains responsive to evolving evidence and the lived experiences of youth with ASD and their families.

#### 1.2.4. Purpose of the Current Study and Research Questions

The purpose of the current study was to better understand the content focus of social–emotional and behavioral IEP goals for local students with ASD, and the quality of those goals as compared to field recommendations. Using a researcher-developed coding tool, which expands prior tools to assess a broader range of quality indicators, this study provides an evaluation of IEP goal quality components for different types of social–emotional and behavioral goals (e.g., pragmatic language, compliance) using a Likert-type scale as opposed to a dichotomous scale (present/not present). This study answered the following research questions:

RQ1: To what extent do social–emotional or behavioral IEP goals for students with ASD include (a) a target behavior, (b) who will evaluate the goal, (c) when the goal will be assessed, (d) the context under which the goal will be assessed, (e) where the goal will be assessed, (f) with whom the student will interact when the goal is assessed, and (g) the mastery criterion to determine how and when the goal will be met?

RQ2: Which of the aforementioned quality indicators are more frequently present in social–emotional or behavioral IEP goals for students with ASD?

RQ3: To what extent are there differences in the proportion of goals with target behaviors that are fully present compared to partially present across each of the different types of target behaviors (e.g., pragmatic language)?

## 2. Materials and Methods

This study was conducted as a part of a larger research–practice partnership, focused on implementation science to improve services and outcomes for students with ASD. Secondary data were de-identified prior to being extracted by the school district partners and there was no personally identifiable information in the dataset. This study was deemed to comprise non-human-subject research by Michigan State University Institutional Review Board (IRB).

### 2.1. Sample and Setting

The sample included 153 total students with ASD in Kindergarten through 12th Grade across 28 schools in the Upper Midwest. The dataset consisted of students with the following characteristics: (1) qualified for special education services under the eligibility category of Autism in the 2022-2023 academic year and (2) had at least one behavioral or social–emotional goal included on their IEP. Goals were coded as behavioral or social–emotional (see Cox et al., 2025, for details), encompassing goals related to a student’s ability to regulate emotions and behaviors and a student’s ability to comprehend, process, and use various modes of communication to facilitate meaningful social exchanges. The sample was predominately male (85.6%) and White (74.5%). Nearly all of the sample (90.8%) received special education services solely for ASD and did not qualify under a secondary eligibility category. See Table 1 for additional sample characteristics.

### 2.2. Measures

The goal quality evaluation tool was created by the research team, expanding on existing validated instruments (e.g., IEP-Q, Ruble et al., 2010b; and R-Gori, Notari, 1988) to provide a more nuanced understanding of the quality of IEP components required in state law (Michigan Department of Special Education, 2026). The tool was monitored for reliability using interobserver agreement ratings. Generalizability was improved by seeking iterative feedback on the tool from ASD experts external to the research team. Every social–emotional and behavioral IEP goal was coded to capture the extent to which each of the following quality indicators were present.

#### 2.2.1. Target Behavior

High-quality IEP goals have clear descriptions of the behavior that is to be targeted for improvement through special education services. The extent to which the target behavior was present, clear, and explicit was coded on a Likert-type scale, where 0 = Not present, 1 = Partially present, and 2 = Fully present. Not present reflected goals in which the target behavior was absent or obscure to the point of being unknown (e.g., “completing a set of activities”). Partially present reflected goals in which the description of the target behavior was vague enough that it could not be replicated by an independent party without additional information (e.g., “improve emotion regulation skills”). Fully present reflected goals in which the target behavior was specific, measurable, and could be replicated by an independent party as written (e.g., increase the use of explicitly identified coping skills). Social/emotional goals focused on pragmatic language or emotional regulation, while behavioral goals were categorized as (a) task completion/engagement, (b) compliance, or (c) executive functioning.

#### 2.2.2. Educator Responsible for Evaluating the Goal

High-quality IEP goals clearly identify which educator is responsible for evaluating whether the IEP goal has been met. The extent to which the educator responsible for evaluating the goals was explicitly identified was coded on a Likert-type scale, where 0 = Not present, 1 = Partially present, and 2 = Fully present. Not present reflected goals in which the educator responsible for evaluating the IEP goal was completely absent. Partially present reflected goals in which the educator responsible was alluded to but was not clearly specified (e.g., “the teacher,” but without indicating whether it was the general education teacher or the special education teacher). Fully present reflected goals in which the educator was explicitly identified (e.g., “the special education teacher”).

#### 2.2.3. Timeline for When the Goal Will Be Assessed

High-quality IEP goals clearly indicate when they will be assessed to determine whether the goal has been met. The extent to which the timeline for when the goal will be assessed was present, clear, and explicit was coded on a Likert-type scale, where 0 = Not present, 1 = Partially present, and 2 = Fully present. Not present reflected goals in which no time frame was mentioned at all. Partially present reflected goals in which the time frame was generically mentioned, but no specific date was identified or could be calculated (e.g., “in the spring”). Fully present reflected goals in which the time frame was specific and clearly identified (e.g., “in 16 weeks”).

#### 2.2.4. Context Under Which the Goal Will Be Assessed

High-quality IEP goals have clear descriptions of the context under which the target behavior will be demonstrated or when the goal will be assessed. The extent to which the context was present, clear, and explicit was coded on a Likert-type scale, where 0 = Not present, 1 = Partially present, and 2 = Fully present. Not present reflected goals in which no context for the target behavior was mentioned or described. Partially present reflected goals in which the context was generically mentioned, but not specific enough to replicate by an independent party (e.g., “when needed”). Fully present reflected goals in which the context was specific, measurable, and could be replicated by an independent party as written (e.g., “during transitions”).

#### 2.2.5. The Location Where the Goal Will Be Assessed

High-quality IEP goals clearly identify where the target behavior will be demonstrated or when the goal will be assessed. The extent to which the location was present, clear, and explicit was coded on a Likert-type scale, where 0 = Not present, 1 = Partially present, and 2 = Fully present. Not present reflected goals in which no location for where the goal will be assessed was mentioned or described. Partially present reflected goals in which the location was generically mentioned, but not specific enough to replicate by an independent party (e.g., “in class,” but it was not clear whether it was the general education classroom or resource room). Fully present reflected goals in which the location was specific, measurable, and could be replicated by an independent party as written (e.g., “in the lunch room”).

#### 2.2.6. With Whom the Student Will Be Interacting When the Goal Is Assessed

High-quality IEP goals clearly identify with whom the student will be interacting when the target behavior is demonstrated or when the goal is assessed. The extent to which the individuals with whom the student will be interacting was present, clear, and explicit was coded on a Likert-type scale, where 0 = Not present, 1 = Partially present, and 2 = Fully present. Not present reflected goals in which there was no indication with whom the student will interact when the target behavior is demonstrated or the goal is assessed. Partially present reflected goals in which the individuals with whom the student would interact were generically mentioned, but it was not specific or explicit (e.g., “in a group”). Fully present reflected goals in which the individuals with whom the student would interact was specific and explicitly identified (e.g., “with non-disabled peers”).

#### 2.2.7. The Mastery Criterion Against Which the Goal Will Be Assessed

High-quality IEP goals clearly identify the criterion against which the goal will be assessed in order to determine whether the goal has been met. The extent to which the mastery criterion was present, clear, and explicit was coded on a Likert-type scale, where 0 = Not present, 1 = Partially present, and 2 = Fully present. Not present reflected goals in which there was no information provided about how the mastery criterion would be measured (e.g., the tool or assessment approach) nor what the mastery criterion were. Partially present reflected goals in which there was information about either the way in which the criterion would be assessed (e.g., measure, tool) or the mastery criterion (e.g., “on 4 out of 5 trials,” but there was no description of what the trials entailed). Fully present reflected goals in which there was information about both the way in which the criterion would be assessed (e.g., measure, tool) and the mastery criterion (e.g., “75% or 3 out of 4 times as evidenced by teacher observation”).

### 2.3. Data Analysis

To answer the first research question, the frequency and percentage of each of the quality indicator codes were calculated. To answer the second research question, the frequencies and percentages were compared across each quality indicator. Finally, the proportion of goals with target behaviors that were fully present and partially present were compared across each of the different types of target behaviors were calculated to answer the third research question. Inferential statistics were not pursued because the data were not normally distributed and the observations of the quality indicators were not independent of each other. Further, analytic approaches that accounted for the nested nature of the data (e.g., students nested within teachers or schools) were not appropriate because there were a very small number of students with ASD in any given classroom, grade level, or school (e.g., a large number of classrooms, grades, and schools only had 1–2 students with ASD).

### 2.4. Procedure

After cleaning the dataset (e.g., removing duplicate students), a codebook was iteratively developed. The codebook included both a priori codes (e.g., pragmatic language, self-injurious behaviors) and in vivo codes (e.g., school information systems). A priori codes were identified based on a review of the literature and best practices serving students with ASD (e.g., Chazin et al., 2025); in vivo codes emerged during the initial coding process until we reached code saturation and no new codes emerged. The research team met weekly to refine the codebook, both collapsing and disaggregating codes until interobserver agreement (IOA) met a minimal threshold of 80%. IOA was calculated as the total number of agreements divided by the total number of disagreements plus agreements, multiplied by 100. To minimize drift, IOA was calculated every 1–2 weeks throughout the coding process. IOA was calculated on a random sample of 44% of students throughout the duration of the coding process (*N* = 68 students, 238 goals). The overall IOA was 86% (range 81–90%). Coding discrepancies were discussed by the full research team until consensus was reached and the final code was recorded.

## 3. Results

**Figure 1:**
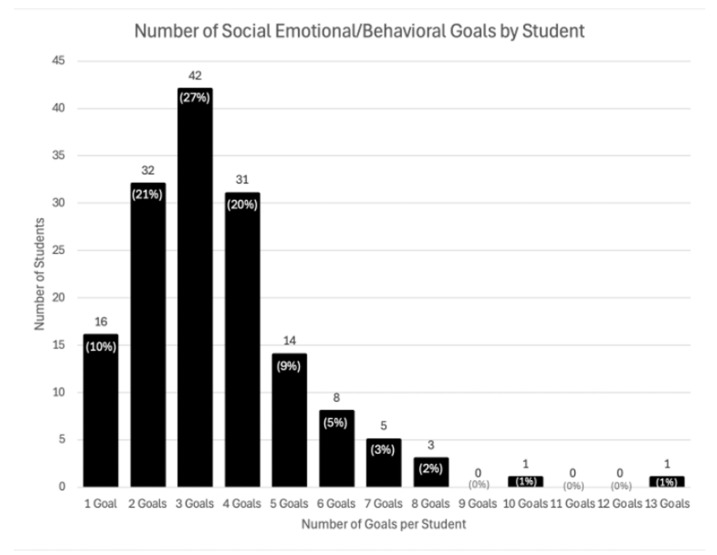
Histogram of Social–Emotional or Behavioral IEP Goals Among Students with ASD.

A total of 530 social–emotional or behavioral IEP goals were coded across 153 autistic students. On average, students had approximately three social–emotional or behavioral IEP goals (see Figure 1). In regard to the first and second research questions, there was variability in the extent to which the quality indicators were present. These results are presented in Table 2. Target behaviors were approximately evenly split between being well operationalized, observable, and measurable (49.62%) and described in the IEP but in such a way that an independent observer would be unable to replicate them (50.19%). Most goals did not indicate which educator would evaluate the goal nor where the goal would be assessed (84% and 75.47%, respectively). Most of the IEP goals included either the measure or tool that would be used to evaluate the goal or the mastery criterion, but not both (91.70%). Of all the quality indicators, the timeline for when the goal would be assessed was the most commonly present (69.81%) and the educator responsible for evaluating the goal was the least commonly present (84.34%).

**Figure 2:**
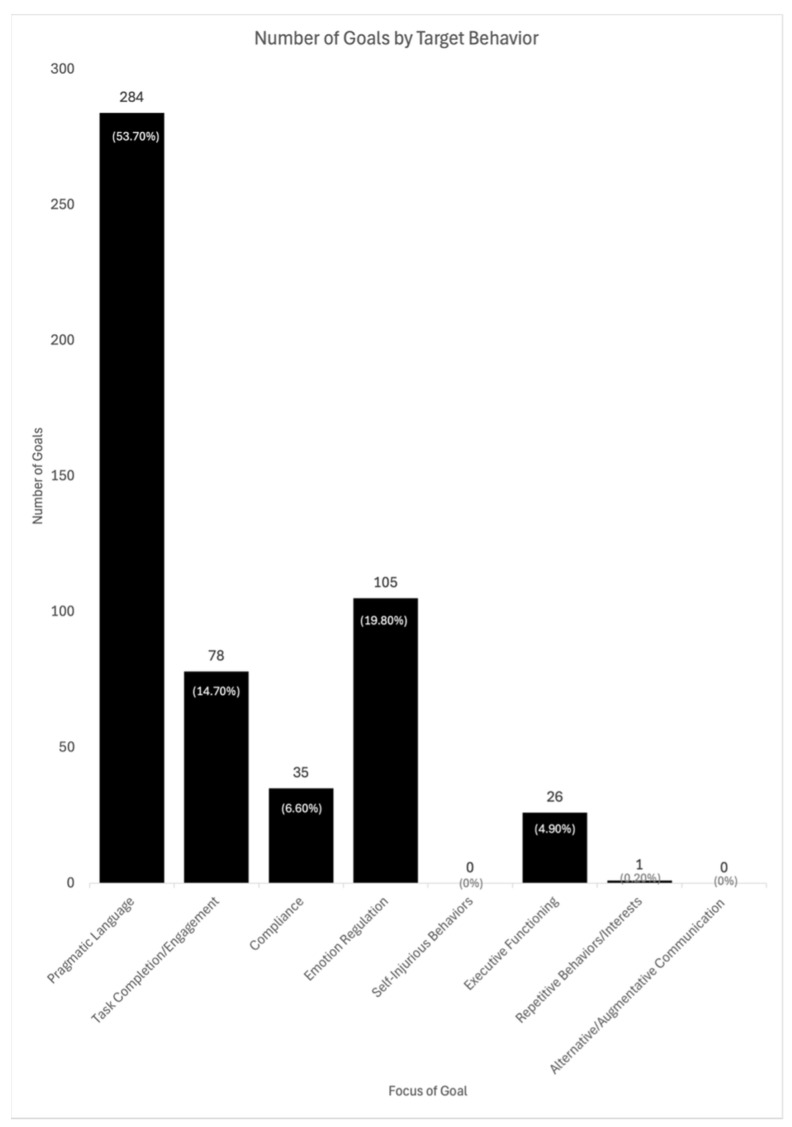
Histogram of the Behaviors Targeted in Social–Emotional or Behavioral IEP Goals.

The IEP goals targeted a variety of behavioral and social–emotional outcomes critical to the treatment of ASD (see Figure 2). The most common target behavior was pragmatic language, followed by emotion regulation, and task completion/engagement. Addressing pragmatic language in IEP goals aligns with some of the defining features of ASD, such as impairment in social interactions and communication. Together, these three target behaviors made up 88.2% of all the behavior and social–emotional goals across all 153 students.

Only a small percentage of goals explicitly and clearly identified the location in which the IEP goal would be evaluated (see Table 3). When indicated, most of the behavioral and social–emotional IEP goals were evaluated in pull-out settings (2.45%), followed by the general education (1.70%) or an unspecified classroom setting (1.70%). Two goals were coded as “other” and both goals indicated they would be assessed in online or virtual contexts (e.g., Google Classroom). These details related to how, when, and where IEP goals will be assessed are key to ensuring students with ASD are being progress monitored in a reliable and valid fashion and may be addressed through systemic IEP goal writing practices.

Finally, in regard to the third research question, task completion/engagement was the most likely to be well operationalized (62.82%), whereas emotion regulation was the least likely to be described in an observable and measurable fashion (36.19%). Table 4 summarizes the extent to which the remaining target behaviors were full or partially present in the IEP goals.

## 4. Discussion

The purpose of this study was to better understand the content focus and quality components of social–emotional and behavioral goals for students with ASD. With an average of 3.46 social–emotional or behavioral goals per student, findings suggest IEP teams recognized the importance of these goals for students with ASD. The greatest number of students had three goals, with most students having between 1 and 5 social–emotional/behavioral goals (88.24%). This quantity is consistent with the average number of social–emotional and behavioral goals identified in previous research (Humbert et al., 2025; McIntyre & Zajic, 2025). Inclusion of social–emotional and behavioral goals for students with ASD is expected given that the diagnostic criteria for ASD include communication and repetitive/restrictive behaviors that would be observed within these categories.

### 4.1. Skills Addressed in Goals

Goals spanned multiple domains of social–emotional and behavioral skills, with pragmatic language skills (*n* = 284, 53.7%) and emotional regulation skills (*n* = 105, 19.8%) appearing most frequently. Notably, priorities identified previously by individuals with ASD and their families (e.g., Chazin et al., 2025), such as self-injurious behaviors (*n* = 0) and alternative/augmented communication modalities (*n* = 0), were absent from this sample. Given the prevalence of self-injurious behaviors and the need for alternative/augmentative communication modalities for students with ASD in schools, it is alarming that there were not any IEP goals addressing these behaviors in the dataset. It should be noted that it is possible programming for these important skills was included in other areas of the IEP (e.g., Behavior Intervention Plans), but they could provide important insights into preventative approaches for collaborating districts. Increasing access to functional communication and reducing self-injurious behaviors are inter-related features of ASD that remain community priorities (Chazin et al., 2025) and should be carefully considered by IEP teams to support access and inclusion in educational settings.

### 4.2. Quality Ratings of Goal Components

Although there may have been quality indicators present in other components of the IEP, results were consistent with prior research (Ruble et al., 2010b; Humbert et al., 2025; McIntyre & Zajic, 2025). The quality components of IEP goals were relatively low with some variability, with less than half of the goals including an observable and measurable target behavior (*n* = 263, 49.62%) and/or the specific and replicable context under which the goal should be performed (*n* = 44.72%). Furthermore, less than 5% of the goals included mastery criterion with both the method to capture student progress (e.g., “as observed by on-time submissions in Google classroom”) and the expected level (e.g., “for at least 4 out of 5 assignments”), leaving IEP teams unable to provide progress monitoring data for more than 95% of the goals. Prior studies have shown elements related to progress monitoring (e.g., who is collecting data and how are data collected) are commonly absent from IEP goals (Goodwin et al., 2022) as well as full IEP documents (e.g., Hott et al., 2021, Humbert et al., 2025; Ruble et al., 2010b), suggesting IEP goals alone are a reasonable estimate of program content. We found similar results, with less than 2% and 5% of goals specifying who was collecting data and how those data were being collected (respectively), suggesting progress monitoring continues to be an area of weakness for social–emotional and behavioral IEP goals among students with ASD. Timely and effective intervention relies on a well-defined, measurable target behavior, and a progress monitoring plan. Systems to support the creation of high-quality goals are essential to ensure IEP teams are able to make meaningful data-based instructional decisions that are connected to improved student outcomes (Christle & Yell, 2010).

Interestingly, most goals included a specific and replicable timeline for when the goal will be assessed (*n* = 237, 69.81%), an observable and measurable target behavior (*n* = 263, 49.62%), and the level of mastery required (*n* = 486, 91.7%). An examination of why these components of the goals may have been more frequently observed was outside the scope of this study, but possible explanations would be (a) the emphasis on these components (i.e., target behavior, timeline, level) within current professional development, (b) the structure of goal templates often include these three elements, and (c) IEP goal banks or examples may be more likely to include these components. Future research should investigate potential reasons for differential quality among goal components to provide responsive suggestions for future research and practice. Structural factors (e.g., compliance-driven practices, IEP templates or mandated systems) may have impacted findings and should be further explored to understand and address potential constraints faced by IEP teams.

Findings suggest social–emotional and behavioral goals for students with ASD do not contain the level of clarity and specificity that would allow them to guide instructional decisions or monitor student progress, although it is possible that more specificity was present in other components of the full IEP document. Given the importance of high quality social–emotional and behavioral goals for students with ASD, combined with consistent and persistent findings of insufficient goals for students with ASD (e.g., Ruble et al., 2010b; Humbert et al., 2025; McIntyre & Zajic, 2025), and their peers (e.g., emotional and behavioral needs; Hott et al., 2021; traumatic brain injury, Goodwin et al., 2022), concentrated efforts to improve goal quality must continue. Without measurable and measured goals, IEPs are unlikely to meet legal requirements or lead to meaningful progress (Christle & Yell, 2010).

### 4.3. Evaluating Goals

This research extends prior work by evaluating goal components using a Likert-type scale rather than a dichotomous score used in previous research (e.g., Ruble et al., 2010b, Humbert et al., 2025, Goodwin et al., 2022). This scaled score allowed us to capture a more nuanced comparison of goal quality, highlighting the goal components completely missing (e.g., who and where the goal will be evaluated) compared to the components with insufficient information (e.g., target behavior, mastery criterion). With this information, future researchers and practitioners can work to include the level of specificity needed to ensure students with ASD receive a high-quality IEP to support meaningful and individualized goals. Additionally, this work provides a comparison of fully present target behaviors by their deductive and in vivo categories. These categories not only represent the localized interpretation of social and emotional areas of need for individuals with ASD but also highlight the possibility that particular types of behaviors (e.g., emotional regulation) may have been more difficult to operationally define. These findings can be used by researchers and practitioners when considering how to define various target behaviors. Intentional selection of instructional practices that are aligned with learning objectives is the first step to providing an Individualized Education Program, making well-defined learning objectives (e.g., IEP goals) imperative to adequate service provisions (Odom et al., 2010).

### 4.4. Limitations and Suggestions for Future Research

This study provides additional information about the content and quality of social–emotional and behavioral goals for students with ASD but should be considered within existing limitations. First, the study utilized a secondary dataset from one regional area within the Upper Midwest. Although the ISD contained multiple local school districts, the sample may not be representative of all Midwestern districts and therefore generalization should be carefully considered. Second, the authors only had access to the student’s IEP goals and not the full IEP document, leaving the possibility that some of the details contributing to high-quality goals could have been reported in other sections and not captured within our dataset, which suggests our findings may underestimate the presence of quality indicators in IEP goals among students with ASD. Third, the researchers remained blinded to all students and teachers, making it impossible to capture insights about the goal writing process and IEP team meetings that could have provided a more nuanced understanding of how and why goals were written the way they were. And fourth, the researchers developed their own goal quality rubric with three levels rather than the traditional dichotomy (present/not present) to serve as a more sensitive measure for comparison. While we engaged in multiple procedures to ensure reliability and content validity, such as an extensive IOA coding process, it is possible the IEP goal quality rubric would not be replicable by other research teams, thereby decreasing the external validity.

### 4.5. Implications for Research

High-quality IEP goals are essential to the provision of special education services for students with disabilities, including those with ASD. Based on findings from this study, teacher preparation programs should incorporate explicit training in supporting speech and language impairments to enhance preservice teacher’s ability to construct IEP goals that help the IEP team make timely and data-informed decisions leading to individualized and responsive interventions in the areas of social–emotional and behavioral skills for students with ASD. Collaboration between schools and families is crucial for fostering the social–emotional well-being of students with disabilities, and actionable frameworks should be included professional development for in-service and preservice teachers. To meet legal obligations and improve long-term outcomes for youth with ASD, administrators and policymakers must recognize the importance of individualized supports within inclusive environments and allocate professional development resources accordingly. This study offers a useful starting point to consider how the content quality of IEP goals may differ across student sociodemographic characteristics (e.g., age, grade level, disability profile) or systems-level processes (e.g., service intensity, available resources,), and the underlying causes for potential differences (e.g., professional learning to support IEP goal writing).

## 5. Conclusions

This study highlights persistent gaps in both the content and quality of social–emotional and behavioral IEP goals for students with ASD, particularly the lack of clarity in progress monitoring and the context specifications. While pragmatic language and emotional regulation were commonly addressed, critical areas aligned with stakeholder-identified priorities, such as self-injurious behaviors and augmentative communication, remained absent. These findings reinforce the urgent need for targeted teacher preparation, family collaboration, and system-wide investment in professional development to ensure that IEPs truly support the complex and evolving needs of students with ASD, enabling them to receive evidence-based support and interventions, in turn achieving meaningful and measurable outcomes during and after school.

## Figures and Tables

**Table 1:** Sample Characteristics (*N* = 153).

Characteristic	*N*	%
Early Elementary	39	23.4%
Kindergarten	12	7.2%
1st Grade	10	6.0%
2nd Grade	17	10.2%
Upper Elementary	52	31.1%
3rd Grade	15	9.0%
4th Grade	15	9.0%
5th Grade	22	13.2%
Middle School	51	30.5%
6th Grade	23	13.8%
7th Grade	15	9.0%
8th Grade	13	7.8%
High School	25	15.0%
9th Grade	10	6.0%
10th Grade	8	4.8%
11th Grade	5	3.0%
12th Grade	2	1.2%
Gender		
Male	131	85.6%
Female	22	14.4%
Race/Ethnicity		
African American or Black	7	4.6%
Asian or Pacific Islander	14	9.2%
White	114	74.5%
Hispanic or Latinx	12	7.8%
Two or More Races	16	10.5%
Native Language		
English	145	94.8%
Not English	8	5.2%
Free or Reduced Meals Status		
Yes	46	29.1%
No	107	69.9%
Secondary Disability Qualification		
None (Autism Only)	139	90.8%
Cognitive Impairment	4	2.6%
Emotional Impairment	2	1.3%
Learning Disability	1	0.6%
Other Health Impairment	4	2.6%
Speech and Language Impairment	2	1.3%
Visual Impairment	1	0.6%

Note. Participants were allowed to select more than one racial/ethnic identity category; thus, percentages may add up to more than 100%.

**Table 2:** Quality of Social–Emotional or Behavioral IEP Goals among Students with ASD.

Quality Indicator	Rating	Number of Goals	% of Total Goals
Target Behavior	Not Present	1	0.19%
	Partially Present	266	50.19%
	Fully Present	263	49.62%
Educator Responsible for Evaluating the Goal	Not Present	447	84.34%
	Partially Present	71	13.40%
	Fully Present	12	2.26%
Timeline for When the Goal will be Assessed	Not Present	14	2.64%
	Partially Present	146	27.55%
	Fully Present	370	69.81%
Context Under Which the Goal will be Assessed	Not Present	226	42.64%
	Partially Present	67	12.64%
	Fully Present	237	44.72%
The Location Where the Goal will be Assessed	Not Present	400	75.47%
	Partially Present	92	17.36%
	Fully Present	38	7.17%
With Whom the Student will be Interacting	Not Present	289	54.53%
	Partially Present	103	19.43%
	Fully Present	138	26.08%
Mastery Criterion	Not Present	20	3.77%
	Partially Present	486	91.70%
	Fully Present	24	4.53%

**Table 3:** Location Where Social–Emotional or Behavioral IEP Goals Were Assessed.

Location	Number of Goals	% of Goals with Location Fully Present	% of Total Goals
General Education Classroom	9	23.7%	1.70%
Pull-Out Setting	13	34.2%	2.45%
Unspecified Classroom	9	23.7%	1.70%
Hallway	0	0.0%	0.0%
Lunch or Recess	1	2.6%	0.19%
Bus	0	0.0%	0.0%
Multiple Locations	4	10.5%	0.75%
Other	2	5.3%	0.38%
Goals with Location Not Fully Present	492	-	92.83%

**Table 4:** Quality of the Description Across Types of Target Behaviors.

Types of Target Behaviors	Partially Present Goals *N* (%)	Fully Present Goals *N* (%)
Pragmatic Language	141 (49.65%)	143 (50.35%)
Task Completion/Engagement	29 (37.18%)	49 (62.82%)
Compliance	14 (40.00%)	21 (60.00%)
Emotional Regulation	67 (63.81%)	38 (36.19%)
Executive Functioning	14 (53.85%)	12 (46.15%)

## Data Availability

The datasets presented in this article are not readily available due to the data-use agreement between the university and intermediate school district. The coding manual will be available in OSF following an embargo from the date of publication to allow for ongoing publications.

## Abbreviations

The following abbreviations are used in this manuscript:

ASD	Autism Spectrum Disorder
IEP	Individual Education Program
